# Waste-Derived NPK Nanofertilizer Enhances Growth and Productivity of *Capsicum annuum* L.

**DOI:** 10.3390/plants10061144

**Published:** 2021-06-04

**Authors:** Heba M. M. Abdel-Aziz, Magda I. Soliman, Aml M. Abo Al-Saoud, Ghada A. El-Sherbeny

**Affiliations:** Botany Department, Faculty of Science, Mansoura University, Mansoura 35516, Egypt; magdaisoliman@yahoo.com (M.I.S.); amlmahmoudgad124@gmail.com (A.M.A.A.-S.); ghada204@mans.edu.eg (G.A.E.-S.)

**Keywords:** *Capsicum annum* L., nanofertilizer, waste recycling, chitosan, foliar fertilizers

## Abstract

Waste generation is a global issue that necessitates effective management for both human and animal health as well as environment. There are several ways to reduce waste, but recycling appears to be the best choice. By recycling, not only will the problem of pollution be resolved, but valuable compounds could be generated to be used as nutrients for plants. In this study, eco-friendly methods were established to produce α- and β-chitosan (CS) (as a source of nitrogen) with different degrees of deacetylation from shrimp shells and squid pin waste, phosphorous through degreasing and calcination of bovine bone and potassium from evaporation of banana peels Kolakhar. The waste bulk products were physically characterized and dry-milled into nano-powders. Different concentrations of the produced nano-NPK fertilizer (10%, 25%, 50% and 100%) were foliar-applied to *Capsicum annum* L. cv. Cordoba plants and compared to commercial chemical fertilizer and untreated control plants. The obtained results revealed that the nano-composite NPK with 25% concentration significantly promoted growth, yield and harvest of *C. annuum* as compared with the control and chemical fertilizer-treated plants. This study demonstrated that the use of an eco-friendly preparation of waste NPK composites, with a low concentration, could be applied as foliar fertilizer over chemical fertilizer to enhance the growth and productivity of *Capsicum*.

## 1. Introduction

There are many threats facing agriculture, including lack of productivity due to pests, global climate change impacts as well as loss of soil fertility [1]. The continuous use of chemical fertilizers (CF) was the basis of traditional farm activities, leading to environmental contamination [2,3]. The global population is projected to grow to 9–10 billion by 2050, meaning an increase of 25–70% of food demand in comparison to current levels [4]. New innovations in agriculture must also be deployed to ensure sustainable development and improve productivity [3,5]. Instead, the world stands up to the challenge of getting rid of solid waste (SW). The lack of management and disposal is causing significant environmental problems, which in turn affect the human health.

During the past decades, the volume of SW produced has been exponentially increased nearly all over the world [6,7]. Great efforts have been achieved in the production of organic fertilizers through SW recycling [7,8,9]. The application of organic fertilizers supplies the needed nutrients for plant growth; hence, it is vital for the development of crop production [10]. Researchers are concerned about farmers’ overuse of CF to satisfy potential demands of the growing world population. Hence, plant mineral nutrition derived from recycled SW is a smart link solving worldwide problems through SW streaming to meet the increased human nutritional needs [11,12].

The nutrients get fixed in soil as insoluble forms and are rendered unavailable to plants due to leaching and decomposition [13]. In order to increase the crop production, renewable, bio- and nano-fertilizers are used for the purpose of protecting the environment and reducing waste [14]. The help of nanotechnology is needed to reduce nutrient losses and raise the yield, as nanoparticles (NPs), i.e., particles with a small dimension (1–100 nm), have ameliorated physicochemical and biological properties such as the large surface area to volume ratio that open the way to substitute the usage of bulk materials with NPs. Thus, it helps in increasing bioavailability and uptake of minerals plus crop yield, decreasing fertilizer wastage, and protecting the environment [15,16,17].

Nanofertilizers (NFs) have emerged as promising alternative smart fertilizers to ensure high crop production and soil restoration. However, there is an urgent need to assess the toxicity of NPs used in agriculture [18,19]. In addition, anything that restricts root growth reduces nutrient uptake [20]. Foliar spray overcomes nutrients limitation in addition to minimizing environmental pollution and improves nutrient utilization by reducing the amounts of fertilizers added to the soil and increasing the crop yield [21]. NPs are easily taken in by the epidermis of leaves and translocated to stems, which facilitates the uptake of active molecules, thus improving growth and productivity of several crop plants [22]. NFs could be synthesized from conventional fertilizer bulk materials after several extraction and purification procedures from using different chemical, physical, mechanical, and biological methods [23,24]. Physical methods, mechanical pressure, ultra-sonication, or electrical energy, etc., depending on the top-down strategy, are cost-effective, low-toxic, eco-friendly, and efficient protocols to fabricate NPs [25].

SW generated must be processed to improve their nutrient content and agricultural benefit in order to contribute to a more biofriendly economy and climate [26]. Seafood SW, i.e., disposal of the fish-processing industry generated with extreme load, are a store of nitrogen source, chitin. Chitin exists in different crystalline forms varying in degree of deacetylation [27,28]. Chitosan (CS), deacetylated chitin, boosts plant growth [29]. Bone meal, a by-product of the rendering industry, is a valuable source of nutrients for plants rich in phosphorous and calcium [30,31]. Hydroxyapatite (HA), demineralized bone, contains no water-soluble P, whereas dicalcium phosphate (DCP), a unique form of fertilizer, is sparingly soluble in water while remaining in a chemical form so that plants can easily access its minerals [32] and is a by-product of direct acidulation of phosphate rock and bones [33,34].

Banana provide significant nutrients that can be recycled as useful plant fertilizer. The potassium content in banana peels is around 40% of the fruit [35]. Potassium carbonate (PC), a fertilizer boosting plant growth, can be isolated from banana pseudo-stem fiber ash obtained after burning of the fiber in the open air [36]. Organic fertilizers provide essential nutrients, thereby improving the crop productivity [37]. Notwithstanding the potential of NPK fertilizer in the initial stage to increase crop growth quickly, organic fertilizers can benefit the plant overall growth and soil organic carbon content in the long term [37]. However, organic fertilizer’s nutrient supply is lower than chemicals, causing the application of organic fertilizers to be reduced, but nano-type fertilizers resolve this constraint [38]. The intensity of organic fertilization is limited by the amount of animal production and/or economic efficiency of production from waste. As shown by the authors of [39], long-term treatment impacts more than the recent single use of mineral or organic fertilizers on emissions of N2O from loamy-sand luvisol. In soil with a higher organic content of carbon, N2O flows outweigh those from soils with a lower organic carbon content following long-term mineral fertilization [39].

The objective of this study was to recycle N, P, and K-rich solid waste into valuable plant fertilizer using a simple, low-cost and eco-friendly plan in pair with a nano-formulation of produced bulk fertilizers as well as to reveal the power of waste-derived nano-NPK fertilizers over traditional chemical fertilizer promoting Capsicum growth and productivity; as one of the world’s major vegetable plants, its quantity and consistency should be increased in this situation.

## 2. Results

### 2.1. Preparation and Charcterization of Waste-Derived Nano NPK Fertilizer

**Figure 1:**
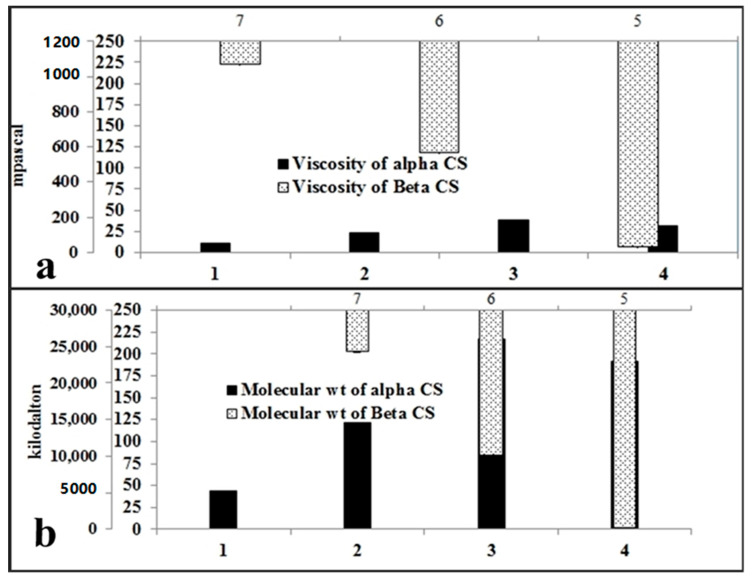
(**a**) Viscosity and (**b**) molecular weight of the prepared α-Cs (1: α-CS1,2: α-CS2,3: α-CS3 and 4: α-CS4) and β-CS (5: β-CS1,6: β-CS2 and 7: β-CS3). Vertical bars represent standard error (±S.E.).

CS structure examination using FT-IR spectroscopy reveals the different characteristic bands for α- and β-CS, as presented in Table S1 and Figure S1A,B. As described in Table 1 and Figure 1, the viscosity and molecular weight are sequenced as follows; α-CS3 > α-CS4 > α-CS2 > α-CS1 and β-CS3 > β-CS2 > β-CS1. The DD % were significantly different and were organized as follows α-CS3 > α-CS4 > α-CS2 > α-CS1 and β-CS2 > β-CS3 > β-CS1. Furthermore, crystallinity and % ash were discursively proportional to the DD % and were ordered as follows: α-CS1 > α-CS2 > α-CS4 > α-CS3 and β-CS1 > β-CS3 > β-CS2, solubility time was inversely proportional to %DDA; the sequence of solubility time was as follows: α-CS1 > α-CS2 > α-CS4 > α-CS3 and β-CS1 > β-CS3 > β-CS2, pH of α- and β- CSs were adjusted at 7.2 for both molecules to be suitable for agricultural use, % yield were significantly different among deacetylation methods, and were arranged as follows α-CS3 > α-CS1 > α-CS4 > α-CS2 and β-CS2 > β-CS3 > β-CS1, the power consumed during the deacetylation operation and the energy cost data were significantly different and were sequenced as follows: α-CS1 > α-CS4 = α-CS3 > α-CS2 and β-CS2 > β-CS3 > β-CS1. SS denoted ~46.16% CaCO_3_, 13.63% protein and 40.21% α-chitin which, in turn, yields ~ 26.65% α-CS that costs ~$13/100 g waste, while SP denoted ~17.88% CaCO_3_, 31.78% protein and 50.34% β-chitin which, in turn, yields ~40.07% β-CS that costs ~$16/100 g waste.

The FT-IR analysis showed all typical absorption characteristics of hydroxyapatite as shown in Table S1 and Figure S1C. The amount of 100 g of raw bovine bone produces about 33.73% pure HA that, in turn, yields 50.22% white, granular odorless, stable, non-flammable powder that is soluble in dilute hydrochloric, nitric and acetic acids 2%, sparingly soluble in water and insoluble in ethanol and costs about $12/100 g waste.

The percentage of carbonate obtained from 10 g dried BP is arranged according to the following sequence: burnt in open air > 500 °C > 600 °C > 400 °C > 300 °C According to FTIR explanations, it is obvious that the resulting fine powder from the open air-ashed Kolakhar is certainly potassium carbonate (Table S1), as it shows the characteristic absorption bands for carbonate as observed in Figure S1D. It was demonstrated that 100 g waste as a potassium rich waste gave 20.44 g of fine dark brown, odorless, non-flammable powder that is instable in air, soluble in water and insoluble in ethanol. This operation costs ≈$0.033/100 g waste.

Some physical characteristics that demonstrated the nano-structure of the prepared nano-waste products were determined. The UV absorption electromagnetic radiation from 220 to 1100 nm for CS resulted in a characteristic sharp absorption band intensity at wavelengths of 245 nm and 243 nm for α- and β-CS NPs, respectively, as observed in Figure S2. These synthesized calcium phosphate nanoparticles showed an absorbance at 241 nm. The optical transmission spectrum for PC was recorded in the wavelength region from 200 to 1100 nm; a unique UV absorption value at 262 nm and 294 nm characteristic for carbonates were observed.

**Figure 2:**
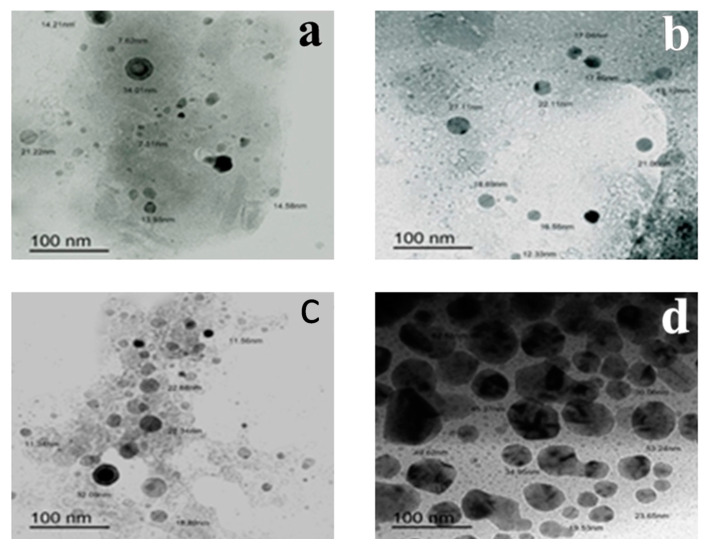
Transmission electron micrographs showing the size variability of (**a**) α-CS NPs, (**b**) β-CSNPs, (**c**) DCP-NPs and (**d**) PC-NPs prepared by the simple dry milling method.

TEM and particle distribution of ground α- and β-CS, PC and DCP showed the success of the dry milling machine in producing a non-homogenous nano-powder of the prepared fertilizers; hexagonal microstructures in the nano range of α-CS and β-CS particles recorded average sizes of 17.41 nm and 14.20 nm. In addition, a dry milling machine results in roughly spherical nano DCP with an average size of 12.71 nm and nano-polygonal K_2_CO_3_ particles with an average size of 28.30 nm as observed in Figure 2 and Table S2.

### 2.2. Growth and Yield Attributes of Treated C. annuum *L.*

**Figure 3:**
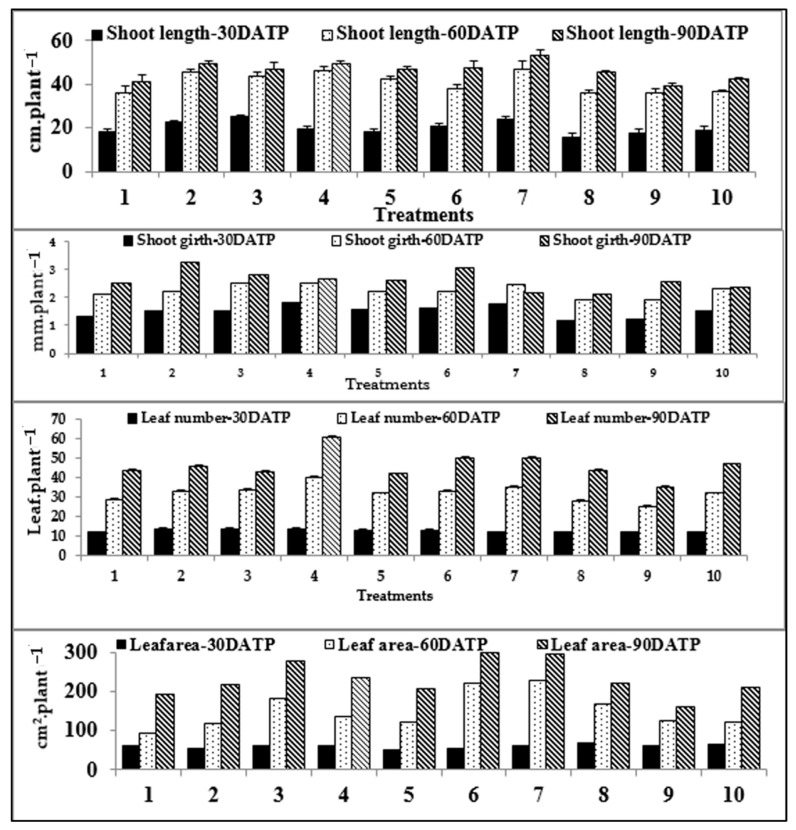
Effect of foliar spray of various levels of waste-derived nano-NPK and chemical fertilizer on shoot length (SL) (cm/plant), shoot girth (SG) (mm/ plant), plant leaves number (LN) and area (LA) in cm^2^/plant of *C. annuum* L. plant at different stages of crop growth. 1: Control, 2: α-nano NPK 10, 3: α-nano NPK 25, 4: α-nano NPK 50, 5: α-nano NPK 100, 6: β-nano NPK 10, 7: β-nano NPK 25, 8: β-nano NPK 50; 9: β-nano NPK 100 and 10: CF. Vertical bars represent standard error (±S.E.).

As compared with the control, the treatment of *Capsicum* with α- and β-NPK nanofertilizers led to significant progression in all growth and developmental attributes determined throughout the growth and developmental stages. Except for higher concentration, the application of foliar fertilizer to *Capsicum* with different concentrations of nano-waste fertilizers and traditional chemical fertilizer (CF) appeared to increase significantly all values of the determined growth attributes, as compared with the control (Figure 3). In Table 2, the growth parameters included in the flowering stage show remarkable changes. As much as 25% of α- and β-NPK fertilizers demonstrated significant progress in flower development time, flower number per plant, decreasing days to fruit set, and increasing its percentages.

As compared with the control, the improvement percentage for the various yield attributes of *Capsicum* in response to foliar application with different concentrations of nano NPK and traditional chemical fertilizers were significant. *Capsicum* treated with β-nano NPK25 stimulated the harvesting stage by −23.40% reduction till reach of harvesting stage as compared with the control as it reached to first harvest after 22 days after anthesis (DAA). The same concentration enhanced the maturity of fruit and so the marketable harvest was attained much faster than in the control with a percentage change of −19.82% (reduction in days compared to control) (Table 2).

On the other hand, chemical foliar-treated *Capsicum* reached a marketable harvest time (MHT) at 60.40 DATP, while the control was delayed to 67.60 DATP. Thus, in this connection and of particular interest, it is interesting to mention that foliar application of NFs resulted in the reduction of the life span of the *Capsicum* crop as compared to the normal life span of the respective crop. As compared with the control, the percent improvement for the various yield attributes of *Capsicum* in response to foliar application with different concentrations of nano-NPK and traditional chemical fertilizers were significantly increased with fruit wt. and seed number/fruit and decreased in post-harvest decay percentage (Table 2).

Foliar addition of α- and β-nano NPK and CF led to significant differences in yield aspects (root, shoot and leaf fresh and dry wt. plus their relative water content) of *Capsicum* as compared to the control (Table 3). In general, high nano-levels had negative impact on these characteristics.

### 2.3. Yield Quality

The *Capsicum* fruit quality attributes were measured during the fruit maturation and harvesting stages as response to foliar-spraying with different levels of nano-waste NPK and traditional chemical fertilizer. Treatments increased the fruit length and width significantly (*p* < 0.05), as compared with the control, while FSI was significantly decreased except in α-nano NPK25-treated *Capsicum* (Table 4). A comparison shows a significant difference (*p* < 0.05) between *Capsicum* treated with different levels of nano-solutions and CF.

The results of mineral composition of *Capsicum* fruits (Table 4) shows that the nitrogen level increased significantly in all treated *Capsicum* when compared to the control. *Capsicum* fruits of the β-nano NPK 25 treatment have the highest phosphorus value (84.01 mg/100 g DW), while fruits are having the lowest phosphorus value (27.67 mg/100 g DW) when treated with β-nano NPK50. *Capsicum* treated with CF, β-nano NPK 10 and 25 and then α-nano NPK25, respectively, have the highest K^+^ content (361.52, 361.31, 358.86 plus 325.67 mg/100 g DW) which was significantly (*p* < 0.05) higher than in the control plants (203.01 mg/100 g DW) (Table 4).

Capsicum sprayed with α and β-nano NPK25 had the highest Ca^2+^ value and Capsicum had the lowest Ca^2+^ value when treated with higher levels of the nano-solution. α-nano NPK 25, β-nano NPK 10 and 25 contain significant (*p* < 0.05) amounts of Cu when compared with other treated *Capsicum* and the control. In addition, the iron content shows that *Capsicum* treated with β-nano NPK25 was the best iron producer (4.113 mg/100 g DW) which was significantly higher than that of other *Capsicum*. As much as 25% of α- and β-nano NPK produces fruits with significant amounts of Zn when compared with any of the treated *Capsicum* and control. The Mn content of CF-*Capsicum* (1.229 mg/100 gDW) was significantly higher than in other treated *Capsicum,* and the control followed small significant changes among other nano-sprayed *Capsicum* (Table 4). The soil physical and chemical characteristics before and after cropping are also listed in Table 4.

**Figure 4:**
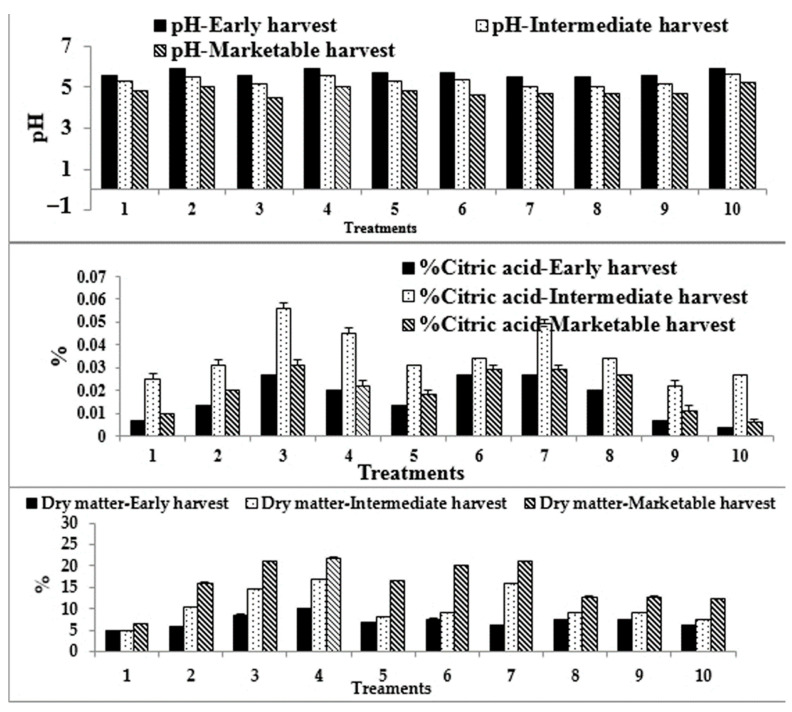
Effect of foliar spray of various levels of waste-derived nano-NPK and chemical fertilizer on pH, % Citric acid, % dry matter at different stages of *C. annuum* fruit maturity. 1: Control, 2: α-nano NPK 10, 3: α-nano NPK 25, 4: α-nano NPK 50, 5: α-nano NPK 100, 6: β-nano NPK 10, 7: β-nano NPK 25, 8: β-nano NPK 50; 9: β-nano NPK 100 and 10; CF. Vertical bars represent standard error (±S.E.).

The pH decreases during ripening and marketable fruits show variable significant values of pH. Except for all treatments, marketable fruits of *Capsicum* treated with CF plus β-nano NPK100 showed significant decreases in % citric acid (Figure 4). Except for α-NPK10 and β-NPK100 nano-levels, all Capsicum marketable fruits showed significant increases in the vitamin C content as compared to the control. The results of the proximate analysis (Table 5 and Figure 4) show that all yield attribute percentages of *Capsicum* were significantly increased (ash content, dry matter, crude fibers, crude fats, crude protein and crude carbohydrates) under the foliar spray with the stated solutions, unlike the moisture content that was significantly decreased if compared with the control value.

## 3. Discussion

Nanofertilizers are modified fertilizers manufactured by chemical, physical or biological methods using nanotechnology to improve their traits and composition, which can enhance the crop productivity [40,41].

CS processing of shrimp shells and squid pins requires many intermittent washing and drying procedures. The method could be industrially viable and environmentally sustainable by decreasing the number of steps in the manufacture of CS [42]. For industrial production, the removal of β-CS may be useful, since smaller reagents and shorter reaction times are required than the crustacean shell production to manufacture α-CS [42].

In various tests, the CS structure analysis with FT-IR spectroscopy shows the different types of α- and β-CS [43,44]. Chitosans with DDA above 70% are favored for their stronger diluted acid solubility [45]. MW dictates the functionality of CS because it significantly influences the physicochemical properties [46]. The variation in Mw is generally influenced by the deacetylation grade difference, the various origins of CS and other variables in CS preparation. α-CS3, β-CS2 and β-CS3 were higher than the recorded MW values [47]. DDA, viscosity, ash content and yield are the four main parameters influencing the preparation efficiency and CS quality. DDA is an important parameter as it affects the solubility, chemical reactivity, and biodegradability. The CS prepared showed higher viscosity values than that reported values in [48,49]. The hydrogen bond between hydroxyl and N-acetyl groups generates the crystallinity of the CS [50]. The crystallinity and DDA exhibit an inverse relationship. α-CS3 and β-CS2 possess the lowest crystallinity and higher DDA. In order to improve its solubility and expand its applications, CS is poorly soluble in water; thus, chemical modification is needed for α- and β-CS which were produced using 600 watt MW for half an hour, followed by one hour autoclave and 30 min in 600 watt MW were chosen as high-quality CS grades. It was developed with a high degree of deacetylation, and its production methods consume less energy while yielding the highest yield when compared to other methods.

The use of BB to generate HA decreases the pollution effect of the waste and transforms it into an extremely valuable product. In the FT-IR spectrum of HA, many bands match with the HA reference spectrum and are in agreement with reported data on HA [51]. When compared on a unit P basis, the DCP was found to be 90–100% as effective as superphosphate in terms of herbage production. The DCP showed circa the same efficiency as the phosphate fertilizer, as did the superphosphate [52,53]. Nano-sized calcium phosphates offer a higher surface area with improved reactivity, a greater drug loading capacity and interaction with the biological environment [54].

PC is an important chemical substance for use in the industrial, pharmaceutical and agricultural sectors. A considerable amount of PCs can be isolated from banana pseudo-stem fiber ash. Kolakhar is quite rich in potassium carbonate, and therefore, can be commercially exploited as a cheap and renewable natural source of potassium carbonate [55]. In this study, the burning of dried peels in open air was the most effective process from which the maximum amount of carbonate can be isolated. This conclusion agrees with data stated by [55].

Farmers have been providing plants with necessary nutrients to get the required yields. Inorganic fertilizers have a poor productivity of nutrients. Another way to improve the production of nutrients and reduce fertilizer depletion is by using NFs [56]. The synthesis of NFs remains in its early stage, with the greatest number of methods employed requiring complex, expensive instruments and low production. In order to replace traditional fertilizers and mitigate the risk of contamination, simplified NF synthesis methods are necessary. To optimize the friction parameters to achieve the optimal nano-size fertilizer at a low price and less time, it is important to understand the effect of the frilling parameters on NFs [57].

*Capsicum annuum* L. is one of the major plants and its antioxidant substances, such as ascorbic acid, flavonoids, phenolic acids and carotenoids, increase the market for these plants. These compounds guard against some cancers, asthma, cardiovascular and neurological conditions [37,58,59].

The conventional way of applying inorganic fertilizers, by means of volatilization, filtration or complex compounds, that are not available for plants, continuously reduces the amount of nutrients utilized. The effects of soil acid, mineral imbalances and groundwater degradation are also harmful to the soil and atmosphere [60]. The plant must then be supplied with nutrients using the latest modern techniques. These methods include adding healthy NPK NFs either by sprinkling or combining the whole vegetative with the soil. NFs are used for quick delivery of nutrients that increase the fertilizer impact duration [61].

The treatments of NFs significantly excelled in boosting of SL, SG, LN and LA of *Capsicum* plants at 30, 60 and 90 DATP compared to CF and the control (Figure 3). This can be because nutrients are quickly applied to the cells by foliar application through stomata, which allows fast transport of nutrients that are vital to plant metabolic processes [62]. It is obvious that high levels of nano-NPK fertilizers showed a negative effect on these attributes. These results agree with those reported in [63].

FT was accelerated by the use of the α-nano NPK25 followed by β-nano NPK25 (Table 2), which might be due to availability of good sunshine and nutrients in the foliar-sprayed leaves with NPs resulting in the accumulation of more photosynthates and induction of early flowering compared to other nano-levels and chemical fertilizer. Foliar spray with α- and β-nano NPK25 improved the flowering developmental stage and maximized the FN more than the control and CF. This could be due to the faster growth and higher number of secondary branches [64]. Foliar spray with high levels of nano-NPK was significantly superior to FN. This result is in agreement with that of [64,65] who worked on carrot and strawberry plants, respectively, and found that spraying plants with nano-liquid fertilizers gave the highest growth parameters.

FW increases with increasing NFs level, then drops at high levels (50 and 100) (Table 2). This may be due to decreases in cell division as a result of cytotoxic effects of the highly concentrated NPs solution [66]. Our results are in contrast with the obtained data [67] which showed that the fruit weight relies on the cultivar and temperature rather than on the culture system (organic or conventional). In addition, only small and non-significant differences between nano-NPK levels and CF in respect to fruit weight were reported by [68,69]. Flaccidity of fruit tissue reduces marketability and consumer acceptance. The increment in PDP during a prolonged period of time could be due to the influence of a high respiration rate, fruit senescence and enzymatic degradation of the fruit’s cell wall [70]. It also may be related to % DM and % MC or the concentration of Ca^2+^ in the fruit’s cell wall [71]. It is apparent that 25% of α- and β-nano NPK fertilizers showed significant positive effects on root, shoot and leaves attributes as compared with the CF and control (Table 3). This may be due to the absorption of NPs by the plant forming organic compounds enhancing the growth. These output results agreed with that reported by [72] on tomato plants.

FL and FW of fruits of α- and β-nano NPK25 were completely different from other treatments and showed higher significant values than the control. The fruit dimensions of *Capsicum* in this study are greater than that reported in [73]. The FSI is an important characteristic of *Capsicum*, as together with the weight, it determines the fruit category [74]. In our experiment, *Capsicum* treated with α-nano NPK25 showed a positive improvement of the FSI. It is notable that MC decreased and DM increased (Table 5 and Figure 4) during *Capsicum* fruit maturity stages and the data recorded are in agreement with report of [58]. These changes may be due to increased respiration rate directed to the formation of valuable compounds during ripening.

Ash content is an index of the mineral content in fruits [75]. A high ash content in a leafy vegetable would suggest a high mineral content, therefore improving the nutritional quality [76]. However, this may not always be the case, as it could be the reverse if it contained toxic metals which also contribute to the percentage of ash content [77]. Fortunately, the heavy metal levels in *Capsicum* fruits as seen in Table 4 were low.

The results of mineral composition of *Capsicum* shown in Table 4 showed that the high ash contents suggest the availability of minerals in treated *Capsicum* which is subsequently confirmed by the considerably high contents of Nitrogen, Phosphorus, Potassium, Ca^2+^, Cu, Fe, Zn and Mn, especially in the 25% α- and β-nano NPK25-sprayed *Capsicum*.

Fiber is a cell wall compound that is usually representative of maturation. The values present in all *Capsicum* plants indicate substantial variations in mature fruit, and the insoluble fiber eventually breaks down into enzymes that are more soluble in order to make the fruit more tender and sweeter [78].

Carbohydrates, the second most abundant component of *Capsicum* (after water), are commonly measured in evaluating the fruit market quality attributes [78,79]. Our results showed that carbohydrate contents are significantly decreasing along with increasing nano-solution levels.

The crude protein content of all treated *Capsicum* was relatively low. This may be the reason that plant sources of protein are of low biological value, as an individual plant source does not contain all the essential amino acids. This agrees with other findings [80,81]. They reported that the protein values recorded for the *Capsicum* varieties are lower than some commonly consumed plant proteins. The same protein values under varied sources of potassium and phosphorous fertilizers were recorded [73].

The most reliable guide to ripening is the titratable acidity (TA). At the beginning of the maturation period, the TA in the handled *Capsicum* declined with fruit maturity. The most abundant organic acid in *Capsicum* is citric acid, and the lack of citric acid from fruit in matured fruit is attributable to a decrease in TA. Due to the activity of the pectinmethylesterase enzyme, increases in the values of TA in the ripening time of fruit may be caused in the course of rift in *Capsicum*, whereas decreases in the TA levels in fruit harvested as a marketable harvest can be due to decreases in organic acids in maturing as they are respired or converted into sugars [82].

Acidity was inversely correlated to pH [83]. The ripe fruit which had a low acid content had a correspondingly higher pH. This acidity may be related to high levels of ascorbic acids formed. Most edible fruits have acidic pH values in the range of 3–5 [84]. The results presented here, in contrast with an earlier report, showed that *Capsicum* juice pH increases and TA decrease with fruit maturity. The pH was lower in the ripe than in the in mature *Capsicum*, as previously reported for other varieties [85].

*Capsicum* is rich in ascorbic acid (vitamin C), a very essential antioxidant [86] and the human body cannot synthesize vitamin C endogenously, so it is an essential dietary component [87]. Vitamin C is instrumental for the neutralization of free radicals that are toxic to the body, iron assimilation, wound healing, collagen building aids the skin, bacterial and viral resistance [88]. In this study, the *Capsicum* studied was found to be an excellent source of ascorbic acid and was in excess of the Recommended Dietary Allowance (RDA) values [89,90]. The ascorbic acid level in *Capsicum* obtained from our research was higher than its level in *Capsicum* obtained from previous research [59]. This may be caused by variations in plant types, soil conditions or climatic conditions [91].

Results regarding the main physical and chemical characteristics and soil major nutrient content before and after the crop cycle are presented in Table 4. The soil pH decreased to a certain extent with different fertilizer treatments [92]. In our study, the soil pH tended to drop in different treatments, it decreased with low nano-NPK levels. This reduction can be explained by the formation of organic acids during organic matter degradation [93].

Soil organic matter (SOM) is a key contributor to soil due to its capacity to affect plant growth indirectly and directly [94]. SOM is widely used as the main parameter for evaluating soil fertility [95]. In general, SOM decreases after the cropping cycle, but increasing the concentration of nano-NPK levels increases their values, which may be due to nano-NPK solution producing enough meal for the *Capsicum* plant to metabolize with little need to mineralization of the SOM, thereby maintaining soil fertility. Examination of the results revealed that there is a significant decrease in all chemicals estimated in soil collected after harvest of foliar-sprayed *Capsicum* by different concentrations of nano-NPK and CF as compared to their values in soil before cropping.

Application of CF and different levels of nano-NPK resulted in a substantial loss of carbonates except in the control and higher concentrations of nano-NPK fertilizers as it shows positive change. This result may be in relation to the maintained organic carbon and organic matter in the soil that belongs to these treatments. This result may be the reason of elevated pH in α-nano NPK100, β-nano NPK50 and 100 as well as in the control. Soil carbonates have been described as an organic matter stabilization agent, mainly due to chemical stabilization mechanisms [96].

The K^+^ concentration deceased as nano-NPK levels increased. It may be due to absorption of a high amount of the ion from soil to maintain vigorous growth, leading to potassium deficient soil [97]. Foliar fertilization of *Capsicum* with different levels of nano-NPK and CF over the control showed a massive decrease in Ca^2+^ concentrations in soil and this may be due to the vital role of Ca^2+^ as a secondary nutrient that is critical to crop development. Ca^2+^ is needed in large amounts by all plants for the formation of cell walls [98]. It is noticeable that a massive decrease in the calcium ion concentration was shown in soil holding *Capsicum* foliar-sprayed with α-nano NPK25. This may explain the high concentration of this ion in fruit which gave it a low post decay harvest percentage.

Fertilization treatments had a negative impact on soil trace metals because large amounts of metals were absorbed during the cropping cycle; higher concentrations of Cu, Fe, Zn and Mn were observed in CF, α-nano NPK 25, β-nano NPK 25 and α-nano NPK10 treatments. These results agree with data reported by [99]. Thus, it is likely that the CF and nano-NPK fertilizers applications improve the soil fertility. However, the effect of waste-derived nano-NPK foliar application on soil fertility was not remarkable as compared to CF.

## 4. Materials and Methods

### 4.1. Materials

Solid waste tested in this study; shrimp shell (SS), squid pins (SP), mature bovine bones (BB) and banana peels (BP) were obtained from Egyptian local markets. Commercial grade chemical NPK fertilizer was obtained from the Al-NASR Fertilizer Company.

### 4.2. Extraction and Preparation of Nanofertilizer from Solid Waste

The amount of 100 g of SS and SP were scraped free of loose tissue, washed with tap water and oven-dried at 70 °C for 2 d. The cleaned waste were blended, demineralized in 1M HCl (1:10, w:v) to remove the minerals (mainly CaCO_3_), deproteinated in boiling 2M NaOH (1:15, w:v) for a time to dissolve proteins, thus isolating the crude chitin, then deacetylated by NaOH 70% (1:15, w:v).

Alpha-CS was obtained by four deacetylation (DA) methods, 1—autoclaving for 8 h at 1.5 atm and 275 °C, 2—incubation in a microwave (MW) with pressure 600 watt for 30 min, 3—incubation in the same microwave for 30 min followed by autoclaving for 1 h at 1.5 atm and 275 °C, and 4—incubation in autoclave at 1.5 atm and 275 °C for 1 h, then in MW for 30 min.

Beta-CS was obtained by three DA methods, 1—autoclaving for 6 h, 2—incubation in the MW for 15 min or by 3—refluxing in the soxhelet apparatus for four hours. The products (α- and β-chitosans) were cooled, washed with d.H_2_O till neutrality, oven-dried at 70 °C, purified using 1% GAA (v:v) and titrated with 2M NaOH. Precipitated CS is washed, oven-dried at low temperature ≈60–70 °C to prevent physical damage in the chain structure [100], then stored in clean dried bottles.

The amount of 100 g of Cow hip, shoulder blade and rib bones of mature bovine (4 years old) were used as raw material for P-source production. The bones were washed with H_2_O to remove impurities, cut into small pieces using a cutter, soaked in a mix of alcohol and acetone to remove the fats and degreased in hot water. Bone fragments were oven-dried, ground, sieved into homogenous fine powder, bleached in 70% NaOH (1:20, w:v) for 30 min, washed till neutrality, mixed with d.H_2_O (1:40, w:v), autoclaved for 1 h [101], filtered and the solid product was washed, oven-dried, then calcined in a muffle furnace at 700 °C for 4 h, bleached again, washed till neutrality, and oven-dried. Acidulation (demineralization) of degreased bones followed by addition of a saturated lime-water solution which precipitated DCP. A mixture of HA and 4% HCl (1:10, w:v) is autoclaved for 2 h. The liquor so obtained is treated with saturated lime-water, a white ppt. of DCP obtained is oven-dried at 60 °C.

Banana peels (BP) (100 g) were used as the material from which potassium carbonate was isolated as a source for K. They were washed with d.H_2_O and dried in an electric furnace at 170 °C for 2 h.

The cleaned peels were oven-dried, burnt at different temperatures. Kholakhar (BP ash: d.H_2_O, 1:20, w:v) was evaporated till reduction in the solution volume to 80 mL, filtered and the supernatant again evaporated to dryness. The solid obtained was kept in a furnace at 250–350 °C for dehydration and then weighed.

The products α- and β-chitosans, HA and K_2_CO_3_ were dry ball milled and subjected to physicochemical analyses for characterization. The suspension of the ball-milled waste- derived NPK nanofertilizer was prepared as follows: 400 ppm N (0.61 g/L CS): 60 ppm P (0.026 g/L DCP): 500 ppm K (0.071 g/L PC) [102] in 0.1% Tween 80 with final pH 5.78. The produced solution was treated as a 100% concentration and further dilutions were made to obtain 10%, 25% and 50% from this stock solution of nanofertilizer both for α- and β-nanofertilizers.

Characterization of Waste-Derived Bulk and Nanofertilizer

(a) Physical characteristics of the chitosans as bulk nitrogen-nutrient sources were measured as viscosity (η) and molecular weight (M_W_); M_W_ was obtained by determining the viscosity [44] of CS solutions at 1% at 30 °C using a Brookfield digital viscometer [103], Mw was calculated using the Mark–Houwink equation: η = KMw^a^. In this CS-solvent system, K = 4.75 × 10^−5^ dL/g and a = 0.72. The degree of deacetylation (DDA) and crystallinity were determined by Fourier Transform Infrared Spectroscopy (FT-IR). The samples were dried and ground with KBr at a sample/KBr ratio of 1:60. Discs were compressed under vacuum. The spectra were recorded at room temperature using a 400 IR instrument (Nicolet, model Impact-410) from 4000 cm^−1^ to 400 cm^−1^. DDA was calculated from the spectra using the equation %DDA = 97.67 − [26.486(A1655/A3450)] [104], while the crystallinity was calculated by equation A1379/A2929 [105]. The pH was determined at the final wash after deacetylation, 1 min after the stirring was initiated. For solubility, 1 mg/mL CS-GAA 1% (*v/v*) solution stirred till a clear solution is attained [106]. The time required in seconds to attain a clear solution is calculated. %Ash and yield; 2 g CS was placed in dry clean crucible, heated in a muffle furnace at 650 °C for 4 h, the crucible was cooled in the furnace and the ash percentage was calculated; %Ash = 100 (weight of residue/sample weight) [107]. The CS yield was calculated per 100 g of dried solid waste [107]. The energy consumption and cost were calculated for each deacetylation process [108],
  E_(kWh/day)_ = P_(W)_ × t_(h/day)_/1000_(W/kW)_
  Cost_(EGP/day)_ = E_(kWh/day)_ × Cost_(Qirsh/kWh)_/100_(qirsh/EGP)_
  Energy (E) in (kW/h) per day, power (P) in watts (W), times number of usage hours per (t), and the cost was measured in cent.
(b) Demineralized HAP (CaHPO_4_) as bulk phosphorous-nutrient source measured characteristics were described as follows: 1—FT-IR analysis: FT-IR spectrum of the HAP in the form of a disc with KBr was recorded using a Mattson 5000 FT-IR spectrophotometer (Unicam, UK) at 25 °C in the range of 400 to 4000 cm^−1^ [106,109]. 2—Yield and Solubility: the dry weight of the product extracted from 100 g of dried samples was applied in the yield equation. Solubility was tested in water, ethanol, 2% HCl and citric acid [110]. 3—Physical attributes: color, odor, state, stability and fire potential were observed and the energy consumption and cost were measured as discussed in part (a).
(c) Potassium carbonate as a bulk potassium-nutrient source was characterized as follows: the carbonates of the ash were burnt at temperatures of 400 °C, 500 °C and 600 °C and ignition in the open air were estimated [111]. Color, odor, state, stability, solubility, fire potential were observed and FT-IR was determined [112]. In addition, the energy consumption and cost were measured as discussed in Part a.
(d) Waste-derived NPK nano fertilizers were initially confirmed by UV-visible spectroscopy using a UV- spectrophotometer (UV-Visible Perkin Elmer Lamda) [112] followed by visualization with transmission electron microscope (TEM) (Jeol-jem-1011, Japan) to observe the morphology and particle size of dry ball-milled particles [113]. In addition, the particle size distribution was statistically studied [114].

### 4.3. Plant Material and Time Course Experiment

For the *Capsicum annuum* L. plant (cv. Cordoba), 15 days-old seedlings were obtained from the Botanical garden of the Faculty of Agriculture, Mansoura University, Egypt. Three experiments were conducted in pots in open field during three seasons (19 March–1 July 2018; 19 March–1 July 2019; 19 March–1 July 2020) in the experimental farm at the Faculty of Science, Mansoura University, Egypt. Each experiment was divided into 13 groups (4 groups for α-nano NPK treatments, 4 groups for β-nano NPK treatments, one group for traditional chemical fertilizer treatment and the remaining groups as water control). Each group contained ten pots spaced by 20 cm holding an equal amount of homogenous soil (5 kg) in which the 15 days old *Capsicum* seedlings were transplanted, irrigated twice a week with tap water. The foliar treatments, as mentioned below, were done three times; the first time after 14 days from transplanting (DATP); the 2nd time was done after 34 DATP and the last foliar treatment was applied at the first harvest time for each treatment.

The scheme of treatments of *Capsicum* was as follows: the first group: 5 pots left without treatment to serve as water control (C), the second group: 5 pots treated with α-nano NPK (10%) (α-nano NPK10); 5 pots treated with α-nano NPK (25%) (α-nano NPK25); 5 pots treated with α-nano NPK (50%) (α-nano NPK50); 5 pots treated with α-nano NPK (100%) (α-nano NPK100), the third group: 5 pots treated with β-nano NPK (10%) (β-nano NPK10); 5 pots treated with β-nano NPK (25%) (β-nano NPK25); 5 pots treated with β-nano NPK (50%) (β-nano NPK50); 5 pots treated with β-nano NPK (100%) (β-nano NPK100) and the fourth group: 5 pots treated with chemical fertilizer (CF).

Once every growth stage (vegetative, flowering and fruiting), in the early morning, using a hand sprayer, the *Capsicum* plants were foliar-sprayed. Effects of treatments were estimated in comparison to CF and control; data (shoot length, shoot girth, leaf number and leaf area) were collected at 30-day intervals over three months of the cropping cycle.

### 4.4. Measurements

Growth and yield attributes, such as the plant height (cm), stem girth (mm), number of leaves/plant, leaf area (cm^2^), days to flowering, number of days for fruit set (DATP), days to marketable harvest (fruits were harvested at the brighten green ripe stage), percentage of fruit set ((number of set of fruitlets/number of flowers at full bloom) × 100), number of branches per plant at 90 DATP, yield per treatment (no.), weight of 6 marketable *Capsicum* fruits (g), average fruit weight (g), no of seeds /fruit, fruit length, fruit width, fruit shape index, post-harvest decay percentage (PDP) (samples having symptoms of chilling injury and diseases were counted but pathogens were not identified), shoot and root fresh weight as well as their dry weight were recorded. Finally, the root length, relative water contents of stem, leaf and roots were estimated [115].

The harvest characteristics were also determined as the following: 1—pH: 25 g of sample were homogenized in a blender, and after the slurry was filtered, the pH was measured with a digital pH-meter [116]. 2—Vitamin C: fruit samples were taken at different harvesting time to determine the vitamin C content in the fruit as mg per 100 g fresh weight by the titration method [117]. 3—Titratable acidity (TA): % citric acid were obtained from fresh tissue (10 g), diluted in distilled water (90 mL), and titrated with NaOH (0.1 N) [118]. %TA = (Titer × 0.1 (NaOH) × 0.67 (malic acid)/1000) × 100). 4—Mineral nutrients: oven-dried leaf and fruit samples were ground to determine mineral contents on a dry weight basis, total nitrogen and phosphorus contents were determined using the Kjeldahl method and colorimetric method using a spectrophotometer, respectively [119]. The potassium and sodium contents were measured using the flame photometer method (JENWAY, PFP-7, ELE Instrument Co. Ltd., UK) [120]. Calcium (Ca), magnesium (Mg), Cupper (Cu), Iron (Fe), zinc (Zn) and Manganese (Mn) were analyzed using atomic absorption spectrophotometer (PERKIN ELMER, 2380 model) [121,122]. 5—Protein content was measured [123,124], the protein contents in g/100 g fruit dry weight were determined by multiplying the nitrogen percentage and protein factor using the formula, %Protein = %Nitrogen × 5.71. 6—%Total fruit moisture: one fruit was taken at different maturity stages and dried in an oven at 60 °C till constant weight, %Moisture = [(fresh wt.-dry wt.)/fresh wt.)] × 100. 7—Ash content was determined by incineration of the residue from moisture determination a 550 °C to constant mass and expressed as g/100 g dry weight [116]. 8—Total carbohydrates were calculated according to equation of %Total carbohydrate = 100 − (% moisture content + % crude protein + % crude lipid + %crude fiber + % ash).

### 4.5. Statistical Analysis

It should be mentioned that all the results obtained in the three experiments were remarkably close, thus only the mean values obtained from the three large-scale experiments will be presented in the corresponding tables and figures given in this study. Experimental data were statistically analyzed using the one-way analysis of variance (ANOVA) with the post hoc Duncan test; *p* value < 0.05 was accepted statistically significant and the analysis was performed using the statistical package for social science for windows (SPSS, version 22).

## 5. Conclusions

Waste is a major problem facing populations worldwide. In this study, we were able to produce a nano-NPK fertilizer from waste materials (shrimp shells, squid pin, bovine bones and banana peels). The application of this nanofertilizer to *Capsicum annuum* leaves proved useful for growth and production. The magnitude of the effects appeared to be concentration-dependent. The 25% concentration of both α- and β-NPK nanofertilizers showed better results with fruit yield and quality. It is recommended to use low concentrations of the produced nanofertilizers. Further toxicological studies are needed to assure that the produced fruits are not toxic to humans and animals.

## Figures and Tables

**Table 1:** Physical properties of alpha and beta CS prepared from natural nitrogen rich waste (shrimp’s exoskeleton and squid pen, respectively); degree of deacetylation of chitin (DD%), crystallinity (a.u.), time consumed (sec) at 1000 rpm to solubilize chitosans in seconds (solubility time), yield (%), energy consumption (kW/h) and energy cost (cent). Means (of three replicates), in each column, followed by similar letter are not significantly different at the 5% probability level using the post hoc Duncan test.

	%DD	Crystallinity	%Ash	Solubility Time	% Yield	Energy Consumption (kW/h)	Energy Cost (Cent)
α-CS1	81.46 a	1.18 c	0.9 c	817 d	26.51 c	16 c	43.30 c
α-CS2	84.92 b	1.12 bc	0.48 b	722 c	24.33 a	0.3 a	0.76 a
α-CS3	86.75 c	0.96 a	0.41 a	580 a	26.65 d	2.3 b	5.60 b
α-CS4	85.48 b	1.05 b	0.45 b	624 b	25.70 b	2.3 b	5.60 b
β-CS1	87.98 a	1.55 c	0.79 c	547 c	33.33 a	12 c	28.99 c
β-CS2	91.35 b	0.57 a	0.35 a	441 a	40.07 c	0.3 a	0.76 a
β-CS3	90.67 b	0.88 b	0.54 b	469 b	36.47 b	4.4 b	10.64 b

α-CS1 = CS prepared by autoclaving for 8 h; α-CS2 = CS prepared by incubation in MW at 600 watt for 30 min; α-CS3 = CS prepared by incubation in MW at 600 for 30 min followed by autoclaving for 1 h; α-CS4 = CS prepared by incubation in autoclave for 1 h, then in the MW at 600 watt for 30 min; β-CS1 = CS prepared by autoclaving for 6 h; β-CS2 = CS prepared by incubated in MW at 600 watt for 30 min and β-CS3 = CS prepared using soxhelet for 4 h.

**Table 2:** Effects of foliar spray of various concentrations of waste-derived nano-NPK and chemical fertilizer on no. of days to 50% of the plants per replicate in blooming (flowering time, FT), no. of flowers per plant (flower/plant, FN), no. of days for fruit set (Fruit set time, FST) (DATP), % fruit set (%FS), days to first harvest (FHT) in DAA, days to marketable harvest (MHT) in DATP, wt. of six marketable fruits (g/fruit) (6FW), average fruit weight (g/fruit, FWav), post-harvest decay percentage (PDP), total yield (g/plant, Yg), (fruit /plant, Yf) and (seeds/ fruit, Ys) of C. *annuum* fruits. Means (of three replicates), in each column, followed by similar letters are not significantly different at the 5% probability level using the post hoc Duncan test.

T	FT	FN	FST	%FS	FHT	MHT	6FW	FWav	PDP	Yg	Yf	Ys
C	39.00 f	8.80 abc	47.00 d	51.33 b	28.00 c	68.00 d	179.00 b	28.25 a	25.81 f	175.17 e	6.20 d	97.35 a
α-nano NPK10	35.00 de	9.20 abc	39.00 c	53.67 b	24.00 b	60.00 c	184.33 c	29.73 b	8.00 c	148.66 d	5.00 c	263.24 d
α-nano NPK25	31.00 a	10.80 c	34.00 a	55.71 b	24.00 b	55.00 a	412.67 h	50.84 i	5.13 b	396.52 j	7.80 e	313.33 e
α-nano NPK50	33.00 bc	9.60 abc	37.00 b	58.43 b	24.00 b	58.00 b	315.33 f	46.30 g	2.78 a	333.38 i	7.20 de	186.79 b
α-nano NPK100	36.00 e	8.40 abc	40.00 c	38.00 ab	24.00 b	61.00 c	178.00 b	30.94 c	15.00 e	123.77 c	4.00 bc	175.07 b
β-nano NPK10	34.00 cd	7.60 ab	37.00 b	52.00 b	23.00 b	58.00 b	259.00 d	40.42 e	2.86 a	282.91 h	7.00 de	230.43 c
β-nano NPK25	33.00 bc	10.00 bc	33.00 a	57.57 b	22.00 a	54.00 a	277.00 e	43.16 f	3.13 a	276.24 g	6.40 d	272.23 d
β-nano NPK50	45.00 g	6.80 a	58.00 e	23.33 a	39.00 e	79.00 e	178.00 b	31.55 d	11.77 d	107.29 b	3.40 ab	107.25 a
β-nano NPK100	46.00 g	6.80 a	58.00 e	25.00 a	33.00 d	79.00 e	170.33 a	30.80 c	15.39 e	80.077 a	2.60 a	200.12 b
CF	36.00 e	9.60 abc	39.00 c	31.33 a	25.00 b	60.00 c	334.66 g	47.94 h	15.79 e	210.94 f	3.80 b	325.66 e

**Table 3:** Effects of foliar spray of various concentrations of waste-derived nano-NPK and traditional chemical fertilizer on number of branches (Branch/plant, BN), root length (cm/plant, RL) at 105 DATP, root fresh weight (RFW), root dry weight (RDW), shoot fresh weight (SFW), shoot dry weight (SDW), leaves fresh weight (LFW) and dry weight (LDW) as well as % relative water content (g/plant, RWC) for each organ of C. *annuum* plants. Means (of three replicates), in each column, followed by similar letter are not significantly different at the 5% probability level using the post hoc Duncan test.

T	BN	RL	RFW	RDW	RWC	
C	2.25 a	22.20 a	2.53 a	1.37 a	36.95 a	
α-nano NPK10	3.00 ab	28.20 ab	5.59 bc	2.58 ab	35.07 a	
α-nano NPK25	2.75 ab	29.10 b	6.05 bc	2.37 ab	52.43 ab	
α-nano NPK50	2.50 ab	27.00 ab	5.14 ab	2.88 b	42.53 ab	
α-nano NPK100	2.75 ab	22.00 a	3.32 ab	1.78 ab	28.97 a	
β-nano NPK10	3.25 ab	25.40 ab	4.75 ab	2.33 ab	33.53 a	
β-nano NPK25	2.75 ab	30.80 b	8.32 c	2.98 b	73.13 b	
β-nano NPK50	3.50 ab	26.30 ab	4.26 ab	1.88 ab	44.62 ab	
β-nano NPK100	3.00 ab	25.80 ab	3.58 ab	1.62 a	33.40 a	
CF	3.75 b	22.30 a	3.75 ab	2.14 ab	27.29 a	
**T**	**SFW**	**SDW**	**RWC**	**LFW**	**LDW**	**RWC**
C	8.20 a	4.63 a	31.33 a	14.59 a	4.30ab	18.79 a
α-nano NPK10	13.80 ab	5.74 abc	56.45 ab	23.92 ab	5.58 ab	25.11 c
α-nano NPK25	19.60 b	6.37 c	108.67 b	33.47 b	6.34 b	18.19 a
α-nano NPK50	15.60 ab	6.31 c	62.02 ab	18.30 ab	3.90 a	19.61 ab
α-nano NPK100	8.40 a	5.07 abc	37.46 a	15.05 a	4.28 ab	18.73 a
β-nano NPK10	16.00 ab	6.35 c	66.39 ab	25.41 ab	5.73 ab	18.86 a
β-nano NPK25	16.60 ab	6.26 bc	69.45 ab	28.82 ab	5.62 ab	41.63 d
β-nano NPK50	9.40 a	4.68 ab	48.55 a	15.07 a	3.83 a	20.44 abc
β-nano NPK100	8.80 a	4.27 a	47.98 a	15.37 a	3.88 a	24.41 bc
CF	10.00 a	5.62 abc	35.60 a	14.85 a	4.90 ab	19.97 abc

**Table 4:** Effects of foliar spray of various concentrations of waste-derived nano-NPK and chemical fertilizer on physical properties (fruit length, cm/fruit (FL), fruit width, cm/fruit (FW) and fruit shape index (FSI) (a.u)) and chemical properties (mineral concentrations (NPK and Ca^2+^) besides trace metals (Cu, Fe, Zn and Mn) of marketable C. *annuum* fruits plus soil physical (PH,E.C) and chemical features (soil organic matter, SOM, carbonates, CO_3_^2−^, N, P and K, Ca^2+^ as well as trace metals (Cu, Fe, Zn and Mn). Means (of three replicates), in each column, followed by similar letter are not significantly different at the 5% probability level using the post hoc Duncan test.

T	Physical Properties			Chemical Properties (mg/100 g DW)								
	FL	FW	FSI	N	P	K^+^	Ca^2+^	Cu	Fe	Zn	Mn	
C	7.88 a	9.76 a	0.81 bc	138.05 a	28.02 a	203.01 a	87.20 a	0.470 a	1.040 a	1.306 b	1.001 a	
α-nano NPK10	8.38 ab	12.56 b	0.67 ab	377.46 c	29.47 a	308.41 b	109.75 ab	0.765 b	2.110 b	2.908 e	1.071 a	
α-nano NPK25	14.81 d	16.48 e	0.90 c	486.40 e	50.40 c	325.67 b	163.70 de	1.826 d	3.685 cd	3.173 f	1.100 ab	
α-nano NPK50	9.54 bc	14.60 bcde	0.65 a	422.76 d	37.18 b	320.75 b	134.13 bc	1.387 c	4.002 d	2.194 d	1.106 ab	
α-nano NPK100	8.88 ab	12.48 b	0.71 ab	390.37 cd	44.10 c	323.40 b	100.73 a	1.364 c	3.497 c	2.271 d	1.099 ab	
β-nano NPK10	8.71 ab	13.14 bc	0.66 ab	489.38 e	58.71 d	361.31 c	144.35 cd	1.803 d	4.030 d	2.909 e	1.106 ab	
β-nano NPK25	10.82 c	15.42 de	0.70 ab	502.12 e	84.01 e	358.86 c	172.82 e	1.777 d	4.113 d	3.112 f	1.095 ab	
β-nano NPK50	9.33 abc	13.68 bcd	0.68 ab	288.76 b	27.67 a	311.51 b	88.91 a	0.632 ab	2.094 b	1.822 c	1.094 ab	
β-nano NPK100	9.02 ab	12.58 b	0.72 ab	281.28 b	29.15 a	207.93 a	88.21 a	0.456 a	1.277 a	0.924 a	1.059 a	
CF	9.47 bc	15.19 cde	0.62 a	404.72 cd	60.78 d	361.52 c	114.50 ab	1.164 c	4.072 d	2.965 e	1.229 b	
Soil												
	**Physical Properties**		**Chemical Properties**									
		**(ds.m^−1^)**	**%**		**ppm**		**Ions mequ./100 g dry soil**		**mg/kg dry soil**			
	**pH**	**E.C.**	**SOM.**	**CO_3_^2−^**	**N**	**P**	**K^+^**	**Ca^2+^**	**Cu**	**Fe**	**Zn**	**Mn**
Before cropping	6.01 f	2.64 b	6.33 k	0.009 g	168.07 k	63.93 k	0.560 i	2.55 k	1.444 i	5.33 g	9.111 d	5.787 k
C	7.34 k	1.54 a	3.20 j	0.010 h	18.10 a	8.05 a	0.114 a	1.49 d	0.005 a	0.681 a	0.091 a	1.005 b
α-nano NPK10	5.82 c	3.85 g	1.96 a	0.003 b	33.02 b	17.04 b	0.215 b	1.62 f	0.052 f	1.408 d	0.542 c	1.956 j
α-nano NPK25	5.67 a	4.60 i	2.00 b	0.002 a	37.03 d	22.09 g	0.260 c	1.08 a	0.064 h	1.372 d	0.565 c	1.815 i
α-nano NPK50	5.86 d	4.62 j	2.08 c	0.005 c	39.07 e	19.11 d	0.292 e	1.28 b	0.052 f	1.687 e	0.421 b	1.768 h
α-nanoNPK100	7.09 j	3.63 e	3.02 i	0.008 f	56.70 f	19.81 e	0.336 f	1.89 j	0.047 d	1.097 c	0.069 a	1.008 c
β-nano NPK10	5.97 e	4.33 h	2.17 e	0.005 d	58.01 g	20.45 f	0.341 f	1.74 g	0.056 g	1.741 f	0.334 b	1.415 g
β-nano NPK25	5.80 b	4.73 k	2.13 d	0.005 d	72.70 j	31.78 h	0.350 g	1.61 e	0.052 f	1.400 d	0.618 c	1.398 f
β-nano NPK50	7.01 h	3.71 f	2.37 g	0.010 h	72.08 i	38.11 i	0.376 h	1.83 h	0.042 c	1.001 b	0.087 a	1.003 a
β-nano NPK100	7.03 i	3.53 d	2.45 h	0.009 g	72.02 h	44.56 j	0.376 h	1.87 i	0.041 b	1.003 b	0.071 a	1.074 e
CF	6.65 g	2.98 c	1.02 b	0.007 e	35.01 c	17.71 c	0.281 d	1.43 c	0.049 e	1.754 f	0.612 c	1.017 d

**Table 5:** % moisture, % ash, % crude fats, % crude fibers, % total carbohydrates, % total proteins (g/100 g DW) and vitamin C (mg/100 g FW) of the harvested C. *annuum* fruits, in response to foliar-sprayed nano-waste fertilizers at different levels and chemical NPK fertilizers. Means (of three replicates), in each column, followed by similar letter are not significantly different at the 5% probability level using the post hoc Duncan test.

T	%Moisture	%Ash	Crude Fats	Crude Fibers	Total Carbohydrates	Total Protein	Vit. C
C	93.58 i	0.93 a	0.69 a	3.03 a	0.86 a	0.91 a	49.75 a
α-nano NPK10	83.86 e	4.12 d	1.38 c	5.57 c	2.59 de	2.49 c	49.75 a
α-nano NPK25	78.94 b	6.63 i	1.64 e	7.11 d	2.48 de	3.21 e	76.00 f
α-nano NPK50	78.11 a	5.21 f	2.77 g	9.36 e	1.76 bc	2.79 d	64.65 e
α-nano NPK100	83.30 d	5.19 f	1.99 f	5.27 c	1.67 bc	2.58 cd	61.97 d
β-nano NPK10	79.85 c	5.85 g	1.07 b	7.36 d	2.64 de	3.23 e	53.52 b
β-nano NPK25	78.90 b	6.05 h	1.475 d	7.43 d	2.83 e	3.31 e	61.97 d
β-nano NPK50	87.10 g	3.57 c	1.68 e	4.19 b	1.55 b	1.91 b	56.34 c
β-nano NPK100	87.60 h	3.20 b	1.65 e	4.18 b	1.52 b	1.86 b	49.75 a
CF	84.81 f	4.34 e	1.98 f	4.06 b	2.13 cd	2.67 cd	87.00 g

## Data Availability

The data presented in this study are available in this article and supplementary material file.

## Supplementary Materials

The following are available online at https://www.mdpi.com/article/10.3390/plants10061144/s1, Figure S1: FT-IR spectra of α-CS (A), β-CS (B), (C) HAP and (D) PC obtained from recycled solid waste, Figure S2: UV-spectra of the prepared nano-waste fertilizers, (A) α- and β-Chitosan, (B) dicalcium phosphate, and (C) Potassium, Table S1: Wave length (cm^−1^) of the main bands obtained for the prepared α- and β-Chitosans and Table S2: Particle size (nm) of prepared waste nano particles.

## References

[1] Do Espirito Santo Pereira A., Caixeta Oliveira H., Fernandes Fraceto L., Santaella C. (2021). Nanotechnology Potential in Seed Priming for Sustainable Agriculture. Nanomaterials.

[2] Zhao L., Lu L., Wang A., Zhang H., Huang M., Wu H., Xing B., Wang Z., Ji R. (2020). Nano-biotechnology in agriculture: Use of nanomaterials to promote plant growth and stress tolerance. J. Agric. Food Chem..

[3] Lowry G.V., Avellan A., Gilbertson L.M. (2019). Opportunities and challenges for nanotechnology in the agri-tech revolution. Nat. Nanotechnol..

[4] Scott N.R., Chen H., Cui H. (2018). Nanotechnology applications and implications of agrochemicals toward sustainable agriculture and food systems. J. Agric. Food Chem..

[5] Kah M., Tufenkji N., White J.C. (2019). Nano-enabled strategies to enhance crop nutrition and protection. Nat. Nanotechnol..

[6] Chen P., Xie Q., Addy M., Zhou W., Liu Y., Wang Y., Cheng Y., Li K., Ruan R. (2016). Utilization of municipal solid and liquid wastes for bioenergy and bioproducts production. Bioresour. Technol..

[7] Abdel-Shafy H.I., Mansour M.S. (2018). Solid waste issue: Sources, composition, disposal, recycling, and valorization. Egypt. J. Pet..

[8] Riber C., Petersen C., Christensen T.H. (2009). Chemical composition of material fractions in Danish household waste. Waste Manag..

[9] Mondal T., Datta J.K., Mondal N.K. (2020). Recycling of municipal solid waste into valuable organic fertilizer towards rejuvenation of crop physiology, yield and soil health. Arch. Agron. Soil Sci..

[10] Yaseen R., Ahmed A.I.S., Mohamed A., Agha M.K.M., Emam T.M. (2020). Nano-fertilizers: Bio-fabrication, application and biosafety. Nov. Res. Microbiol. J..

[11] Wang F., Han W., Chen S., Dong W., Qiao M., Hu C., Liu B. (2020). Fifteen-year application of manure and chemical fertilizers differently impacts soil ARGs and microbial community structure. Front. Microbiol..

[12] Dueñas M., García-Estévez I. (2020). Agricultural and Food Waste: Analysis, Characterization and Extraction of Bioactive Compounds and Their Possible Utilization. Foods.

[13] Alshaal T., El-Ramady H. (2017). Foliar application: From plant nutrition to biofortification. Environ. Biodivers. Soil Secur..

[14] Li R., Tao R., Ling N., Chu G. (2017). Chemical, organic and bio-fertilizer management practices effect on soil physicochemical property and antagonistic bacteria abundance of a cotton field: Implications for soil biological quality. Soil Tillage Res..

[15] Tarafdar J., Raliya R., Mahawar H., Rathore I. (2014). Development of zinc nanofertilizer to enhance crop production in pearl millet (*Pennisetum americanum*). Agric. Res..

[16] Dimkpa C.O., Bindraban P.S. (2016). Fortification of micronutrients for efficient agronomic production: A review. Agron. Sustain. Dev..

[17] Tapan A., Biswas A., Kundu S. (2010). Nano-fertiliser-a new dimension in agriculture. Indian J. Fertil..

[18] Zulfiqar F., Navarro M., Ashraf M., Akram N.A., Munné-Bosch S. (2019). Nanofertilizer use for sustainable agriculture: Advantages and limitations. Plant Sci..

[19] Achari G.A., Kowshik M. (2018). Recent developments on nanotechnology in agriculture: Plant mineral nutrition, health, and interactions with soil microflora. J. Agric. Food Chem..

[20] Morab P.N., Sumanth Kumar G., Akshay K. (2021). Foliar nutrition of nano-fertilizers: A smart way to increase the growth and productivity of crops. J. Pharmacogn. Phytochem..

[21] Hussain S., Mumtaz M., Manzoor S., Shuxian L., Ahmed I., Skalicky M., Brestic M., Rastogi A., Ulhassan Z., Shafiq I. (2021). Foliar application of silicon improves growth of soybean by enhancing carbon metabolism under shading conditions. Plant Physiol. Biochem..

[22] Malerba M., Cerana R. (2016). Chitosan effects on plant systems. Int. J. Mol. Sci..

[23] Qureshi A., Singh D., Dwivedi S. (2018). Nano-fertilizers: A novel way for enhancing nutrient use efficiency and crop productivity. Int. J. Curr. Microbiol. Appl. Sci.

[24] Arole V., Munde S. (2014). Fabrication of nanomaterials by top-down and bottom-up approaches-an overview. J. Mater. Sci.

[25] Satyanarayana T., Sudhakar R. (2018). A Review on Chemical and Physical Synthesis Methods of Nanomaterials. Int. J. Res. Appl. Sci. Eng. Technol..

[26] Silva Y.d.S., Naval L.P. (2018). Segregation of solid waste from a fish-processing industry: A sustainable action. Rev. Ambiente Água.

[27] Cristóvao R.O., Botelho C.M., Martins R.J., Loureiro J.M., Boaventura R.A. (2015). Fish canning industry wastewater treatment for water reuse–a case study. J. Clean. Prod..

[28] Aam B.B., Heggset E.B., Norberg A.L., Sørlie M., Vårum K.M., Eijsink V.G. (2010). Production of chitooligosaccharides and their potential applications in medicine. Mar. Drugs.

[29] Li R., He J., Xie H., Wang W., Bose S.K., Sun Y., Hu J., Yin H. (2019). Effects of chitosan nanoparticles on seed germination and seedling growth of wheat (*Triticum aestivum* L.). Int. J. Biol. Macromol..

[30] Garcia R.A., Rosentrater K.A. (2008). Concentration of key elements in North American meat & bone meal. Biomass Bioenergy.

[31] Kivelä J., Chen L., Muurinen S., Kivijärvi P., Hintikainen V., Helenius J. (2015). Effects of meat bone meal as fertilizer on yield and quality of sugar beet and carrot. Agric. Food Sci..

[32] Cabeza L.F., Castell A., Barreneche C.d., De Gracia A., Fernández A. (2011). Materials used as PCM in thermal energy storage in buildings: A review. Renew. Sustain. Energy Rev..

[33] Trinh H.X., Truong N.X., Van Dung N., Van Trinh V. (2017). Production of dicalcium phosphate from lao cai second grade apatite ore leached by dilute hydrochloric acid. Vietnam. J. Sci. Technol..

[34] Brennan R., Bolland M. (2005). Effectiveness of dicalcium phosphate compared with superphosphate for wheat grown on acidic sandy soils. J. Plant Nutr..

[35] Hussein H., Shaarawy H., Hussien N.H., Hawash S. (2019). Preparation of nano-fertilizer blend from banana peels. Bull. Natl. Res. Cent..

[36] Abdullah N., Sulaiman F., Miskam M.A., Taib R.M. (2014). Characterization of banana (*Musa* spp.) pseudo-stem and fruit-bunch-stem as a potential renewable energy resource. Int. J. Biol. Vet. Agric. Food Eng..

[37] Liu Q., Meng X., Li T., Raza W., Liu D., Shen Q. (2020). The growth promotion of peppers (*Capsicum annuum* L.) by Trichoderma guizhouense NJAU4742-based biological organic fertilizer: Possible role of increasing nutrient availabilities. Microorganisms.

[38] Qiu M., Zhang R., Xue C., Zhang S., Li S., Zhang N., Shen Q. (2012). Application of bio-organic fertilizer can control Fusarium wilt of cucumber plants by regulating microbial community of rhizosphere soil. Biol. Fertil. Soils.

[39] Sosulski T., Szara E., Szymańska M., Stępień W. (2017). N_2_O emission and nitrogen and carbon leaching from the soil in relation to long-term and current mineral and organic fertilization-a laboratory study. Plant Soil Environ..

[40] Al-Juthery H., Ali N., Al-Taey D., Ali E. (2018). The impact of foliar application of nanaofertilizer, seaweed and hypertonic on yield of potato. Plant Arch..

[41] Elemike E.E., Uzoh I.M., Onwudiwe D.C., Babalola O.O. (2019). The role of nanotechnology in the fortification of plant nutrients and improvement of crop production. Appl. Sci..

[42] Jung J., Zhao Y. (2011). Characteristics of deacetylation and depolymerization of β-chitin from jumbo squid (*Dosidicus gigas*) pens. Carbohydr. Res..

[43] Chen S.-C., Wu Y.-C., Mi F.-L., Lin Y.-H., Yu L.-C., Sung H.-W. (2004). A novel pH-sensitive hydrogel composed of N, O-carboxymethyl chitosan and alginate cross-linked by genipin for protein drug delivery. J. Control. Release.

[44] Agarwal M., Agarwal M.K., Shrivastav N., Pandey S., Das R., Gaur P. (2018). Preparation of chitosan nanoparticles and their in-vitro characterization. Int. J. Life Sci. Sci. Res..

[45] Heidari F., Razavi M., Bahrololoom M.E., Tahriri M., Rasoulianboroujeni M., Koturi H., Tayebi L. (2018). Preparation of natural chitosan from shrimp shell with different deacetylation degree. Mater. Res. Innov..

[46] Czechowska J., Zima A., Paszkiewicz Z., Lis J., Ślósarczyk A. (2014). Physicochemical properties and biomimetic behaviour of α-TCP-chitosan based materials. Ceram. Int..

[47] Zhao Y., Ju W.-T., Jo G.-H., Jung W.-J., Park R.-D., Elnashar M. (2011). Perspectives of chitin deacetylase research. Biotechnology of Biopolymers.

[48] El-Sayed S.M., El-Beltagy A.E. (2011). Effect of extraction processing sequences on the physicochemical and functional properties of shrimp chitosans. Assiut J. Agric. Sci..

[49] Cho Y.I., No H.K., Meyers S.P. (1998). Physicochemical characteristics and functional properties of various commercial chitin and chitosan products. J. Agric. Food Chem..

[50] de Alvarenga E.S. (2011). Characterization and properties of chitosan. Biotechnol. Biopolym..

[51] Labuda M., Saeid A., Chojnacka K., Górecki H. (2012). Zastosowanie Bacillus megaterium w solubilizacji fosforu. Przemysł Chem..

[52] Edmeades D. (2000). The agronomic effectiveness of lime-reverted and dicalcic superphosphates: A review. N. Zeal. J. Agric. Res..

[53] Hagin J., Katz S. (1985). Effectiveness of partially acidulated phosphate rock as a source to plants in calcareous soils. Fertil. Res..

[54] Vecbiskena L., Gross K.A., Riekstina U., Yang T.C.-K. (2015). Crystallized nano-sized alpha-tricalcium phosphate from amorphous calcium phosphate: Microstructure, cementation and cell response. Biomed. Mater..

[55] Deka D.C., Talukdar N.N. (2007). Chemical and spectroscopic investigation of Kolakhar and its commercial importance. Ind. J. Tradit. Knowl..

[56] Dhewa T. (2015). Nanotechnology applications in agriculture: An update. Octa J. Environ. Res..

[57] Das D., Bhutia Z.T., Chatterjee A., Banerjee M. (2019). Mechanochemical Pd (II)-catalyzed direct and C-2-selective arylation of indoles. J. Org. Chem..

[58] Rahman M., Halim G., Chowdhury M., Hossain M., Rahman M. (2014). Changes in physicochemical attributes of sweet pepper (*Capsicum annum* L.) during fruit growth and development. Bangladesh J. Agric. Res..

[59] Nerdy N. (2018). Determination of vitamin C in various colours of bell pepper (*Capsicum annuum* L.) by Titration Method. Alchemy J. Penelit. Kim..

[60] Stewart W., Dibb D., Johnston A., Smyth T. (2005). The contribution of commercial fertilizer nutrients to food production. Agron. J..

[61] Naderi M., Danesh-Shahraki A. (2013). Nanofertilizers and their roles in sustainable agriculture. Int. J. Agric. Crop Sci..

[62] Rajasekar M., Udhaya Nandhini D., Suganthi S. (2017). Supplementation of mineral nutrients through foliar spray-A review. Int. J. Curr. Microbiol. Appl. Sci..

[63] Elshamy M.T., Husseiny S.M., Farroh K.Y. (2019). Application of nano-chitosan NPK fertilizer on growth and productivity of potato plant. J. Sci. Res. Sci..

[64] Kumar U.J., Bahadur V., Prasad V., Mishra S., Shukla P. (2017). Effect of different concentrations of iron oxide and zinc oxide nanoparticles on growth and yield of strawberry (*Fragaria x ananassa* Duch) cv. Chandler. Int. J. Curr. Microbiol. Appl. Sci..

[65] Elizabath A., Bahadur V., Misra P., Prasad V.M., Thomas T. (2017). Effect of different concentrations of iron oxide and zinc oxide nanoparticles on growth and yield of carrot (*Daucus carota* L.). J. Pharmacogn. Phytochem..

[66] Madkour L.H. (2020). Nanotoxicity, Cytotoxicity, and Genotoxicity Mechanisms of Nanomaterials. Nanoparticles Induce Oxidative and Endoplasmic Reticulum Stresses.

[67] Abu-Zahra T., Al-Ismail K., Shatat F. (2006). Effect of organic and conventional systems on fruit quality of strawberry (fragaria× ananassa duch) grown under plastic house conditions in the Jordan Valley. Proceedings of the I International Symposium on Fresh Food Quality Standards: Better Food by Quality and Assurance 741.

[68] Al-Antary T.M., Kahlel A.-M.S., Ghidan A.Y., Asoufi H.M. (2020). Effects of nanotechnology liquid fertilizers on fruit set and pods of broad bean (vicia faba L.). Fresen. Environ. Bull.

[69] Ghidan A.Y., Al-Antary T.M., Awwad A.M., Ayad J.Y. (2018). Physiological effect of some nanomaterials on pepper (*Capsicum annuum* L.) plants. Fresenius Environ. Bull..

[70] Ciccarese A., Stellacci A.M., Gentilesco G., Rubino P. (2013). Effectiveness of pre-and post-veraison calcium applications to control decay and maintain table grape fruit quality during storage. Postharvest Biol. Technol..

[71] Hazard B., Zhang X., Colasuonno P., Uauy C., Beckles D.M., Dubcovsky J. (2012). Induced mutations in the starch branching enzyme II (SBEII) genes increase amylose and resistant starch content in durum wheat. Crop. Sci..

[72] Kalbani F.O.S.A., Salem M.A., Cheruth A.J., Kurup S.S., Senthilkumar A. (2016). Effect of some organic fertilizers on growth, yield and quality of tomato (*Solanum lycopersicum*). Int. Lett. Nat. Sci..

[73] Hegazi A.M., El-Shraiy A.M., Ghoname A. (2017). Growth, yield and nutritional quality of sweet pepper plants as affected by potassium and phosphate fertilizers varying in source and solubility. Curr. Sci. Int..

[74] Navarro J., Garrido C., Carvajal M., Martinez V. (2002). Yield and fruit quality of pepper plants under sulphate and chloride salinity. J. Hortic. Sci. Biotechnol..

[75] Esayas K., Shimelis A., Ashebir F., Negussie R., Tilahun B., Gulelat D. (2011). Proximate composition, mineral content and antinutritional factors of some capsicum (*Capsicum annum*) varieties grown in Ethiopia. Bull. Chem. Soc. Ethiop..

[76] Aletan U., Kwazo H. (2019). Analysis of the Proximate Composition, Anti-Nutrients and Mineral Content of Maerua Crassifolia Leaves. Niger. J. Basic Appl. Sci..

[77] Ukam N.U. (2008). The potentials of some lesser known vegetables. Niger. J. Nutr. Sci..

[78] Bernardo A., Martinez S., Alvarez M., Fernandez A., Lopez M. (2008). The composition of two Spanish pepper varieties (Fresno de la Vega and Benavente-Los Valles) in different ripening stages. J. Food Qual..

[79] Martínez S., Curros A., Bermúdez J., Carballo J., Franco I. (2007). The composition of Arnoia peppers (*Capsicum annuum* L.) at different stages of maturity. Int. J. Food Sci. Nutr..

[80] Emmanuel-Ikpeme C., Henry P., Okiri O.A. (2014). Comparative evaluation of the nutritional, phytochemical and microbiological quality of three pepper varieties. J. Food Nutr. Sci..

[81] Aremu M., Nweze C., Alade P. (2011). Evaluation of protein and amino acid composition of selected spices grown in the middle belt region of Nigeria. Pak. J. Nutr..

[82] Anthon G.E., Barrett D.M. (2012). Pectin methylesterase activity and other factors affecting pH and titratable acidity in processing tomatoes. Food Chem..

[83] Garcia E., Barrett D.M. (2006). Evaluation of processing tomatoes from two consecutive growing seasons: Quality attributes, peelability and yield. J. Food Process. Preserv..

[84] Akbudak B. (2010). Effects of harvest time on the quality attributes of processed and non-processed tomato varieties. Int. J. Food Sci. Technol..

[85] Estrada B., Bernal M.A., Díaz J., Pomar F., Merino F. (2000). Fruit development in capsicum a nnuum: Changes in capsaicin, lignin, free phenolics, and peroxidase patterns. J. Agric. Food Chem..

[86] Igwemmar N., Kolawole S., Imran I. (2013). Effect of heating on vitamin C content of some selected vegetables. Int. J. Sci. Technol. Res..

[87] Li Y., Schellhorn H.E. (2007). New developments and novel therapeutic perspectives for vitamin C. J. Nutr..

[88] Medina-Juárez L.Á., Molina-Quijada D.M., Sánchez C.L.D.T., González-Aguilar G.A., Gámez-Meza N. (2012). Antioxidant activity of peppers (*Capsicum annuum* L.) extracts and characterization of their phenolic constituents. Interciencia.

[89] Osuna-García J.A., Wall M.M., Waddell C.A. (1998). Endogenous levels of tocopherols and ascorbic acid during fruit ripening of New Mexican-type chile (*Capsicum annuum* L.) cultivars. J. Agric. Food Chem..

[90] Howard L., Talcott S., Brenes C., Villalon B. (2000). Changes in phytochemical and antioxidant activity of selected pepper cultivars (*Capsicum* species) as influenced by maturity. J. Agric. Food Chem..

[91] Aćamović-Đoković G., Pavlović R., Mladenović J., Đurić M. (2011). Vitamin C content of different types of lettuce varieties. Acta Agric. Serbica.

[92] Dong W., Zhang X., Wang H., Dai X., Sun X., Qiu W., Yang F. (2012). Effect of different fertilizer application on the soil fertility of paddy soils in red soil region of southern China. PLoS ONE.

[93] Obalum S., Chibuike G., Peth S., Ouyang Y. (2017). Soil organic matter as sole indicator of soil degradation. Environ. Monit. Assess..

[94] Lee S.B., Lee C.H., Jung K.Y., Do Park K., Lee D., Kim P.J. (2009). Changes of soil organic carbon and its fractions in relation to soil physical properties in a long-term fertilized paddy. Soil Tillage Res..

[95] Huang Y., Sun W. (2006). Changes in topsoil organic carbon of croplands in mainland China over the last two decades. Chin. Sci. Bull..

[96] Sayara T., Basheer-Salimia R., Hawamde F., Sánchez A. (2020). Recycling of Organic Wastes through Composting: Process Performance and Compost Application in Agriculture. Agronomy.

[97] Mažeika R., Arbačiauskas J., Masevičienė A., Narutytė I., Šumskis D., Žičkienė L., Rainys K., Drapanauskaite D., Staugaitis G., Baltrusaitis J. (2020). Nutrient Dynamics and Plant Response in Soil to Organic Chicken Manure-Based Fertilizers. Waste Biomass Valorization.

[98] Farhat N., Vrouwenvelder J.S., Van Loosdrecht M.C., Bucs S.S., Staal M. (2016). Effect of water temperature on biofouling development in reverse osmosis membrane systems. Water Res..

[99] Nasr M., Mahmoud A.E.D., Fawzy M., Radwan A. (2017). Artificial intelligence modeling of cadmium (II) biosorption using rice straw. Appl. Water Sci..

[100] Toan N.V. (2009). Production of chitin and chitosan from partially autolyzed shrimp shell materials. Open Biomater. J..

[101] Ho C.H., Cacace J.E., Mazza G. (2007). Extraction of lignans, proteins and carbohydrates from flaxseed meal with pressurized low polarity water. LWT Food Sci. Technol..

[102] Abdel-Aziz H., Hasaneen M.N., Omar A. (2018). Effect of foliar application of nano chitosan NPK fertilizer on the chemical composition of wheat grains. Egypt. J. Bot..

[103] Mirzadeh H., Yaghoubi N., Amanpour S., Ahmadi H., Mohagheghi S., Hormozi F. (2002). Preparation of chitosan derived from shrimp’s shell of Persian Gulf as a blood hemostasis agent. Iran. Polym. J..

[104] Monteiro O.A., Airoldi C. (2005). The influence of chitosans with defined degrees of acetylation on the thermodynamic data for copper coordination. J. Colloid Interface Sci..

[105] Agarwal M., Agarwal M.K., Shrivastav N., Pandey S., Gaur P. (2018). A simple and effective method for preparation of chitosan from chitin. Int. J. Life. Sci. Sci. Res.

[106] Ahmed A.S., Hassan A.M., Nour M.H. (2021). Utilization of chitosan extracted from shrimp shell waste in wastewater treatment as low cost biosorbent. Egypt. J. Chem..

[107] Ul-Islam M., Shah N., Ha J.H., Park J.K. (2011). Effect of chitosan penetration on physico-chemical and mechanical properties of bacterial cellulose. Korean J. Chem. Eng..

[108] Mukherjee S.K. (1981). Energy policy and planning in India. Energy.

[109] Zhang J., Kamdem D.P. (2000). FTIR characterization of copper ethanolamine—wood interaction for wood preservation. Holzforschung.

[110] Dorozhkin S.V., Epple M. (2002). Biological and medical significance of calcium phosphates. Angew. Chem. Int. Ed..

[111] Sadek M. (2015). Impact of the Invasive Ipomoea carnea Jacq. on Plant Diversity Along the Canal and Drain Banks of Nile Delta, Egypt. Catrina: Int. J. Environ. Sci..

[112] Stanienda-Pilecki K.J. (2019). The importance of fourier transform infrared spectroscopy in the identification of carbonate phases differentiated in magnesium content. Spectroscopy.

[113] Estrada-Urbina J., Cruz-Alonso A., Santander-González M., Méndez-Albores A., Vázquez-Durán A. (2018). Nanoscale zinc oxide particles for improving the physiological and sanitary quality of a Mexican landrace of red maize. Nanomaterials.

[114] Caputo F., Clogston J., Calzolai L., Rösslein M., Prina-Mello A. (2019). Measuring particle size distribution of nanoparticle enabled medicinal products, the joint view of EUNCL and NCI-NCL. A step by step approach combining orthogonal measurements with increasing complexity. J. Control. Release.

[115] Thakur G., Singh A., Maurya P.K., Patel P., Kumar U. (2018). Effect of plant spacing on growth, flowering, fruiting and yield of Capsicum (*Capsicum annuum* L) hybrid buffalo under natural ventilated polyhouse. J. Pharmaco. Phytochem.

[116] Kalra Y.P. (1995). Determination of pH of soils by different methods: Collaborative study. J. Aoac Int..

[117] Sohair E.E., Abdall A.A., Amany A.M., Houda R.A. (2018). Effect of nitrogen, phosphorus and potassium nano fertilizers with different application times, methods and rates on some growth parameters of Egyptian cotton (*Gossypium barbadense* L.). Bioscience Research.

[118] Turhan A., Ozmen N., Serbeci M., Seniz V. (2011). Effects of grafting on different rootstocks on tomato fruit yield and quality. Hortic. Sci..

[119] Cottenie A. (1980). Soil and Plant Testing as a Basis of Fertilizer Recommendations. FAO Soils Bull..

[120] Chapman H., Pratt P. (1982). Methods of Plant Analysis. I. Methods of Analysis for Soils, Plants and Water.

[121] FAO/UNDP Study Tour to the People’s Republic of China (1980). China: The Agricultural Training System: Report on an FAO/UNDP Study Tour to the People’s Republic of China 5 October to 2 November 1978.

[122] Allen S.E., Grimshaw H.M., Parkinson J.A., Quarmby C. (1974). Chemical Analysis of Ecological Materials.

[123] Mannan M. (2014). Foliar and soil fertilization effect on seed yield and protein content of soybean. Bangladesh Agron. J..

[124] Peach K., Tracey M.V. (1955). Modern Methods of Plant Analysis.

