# Supplementary material for: Waste-Derived NPK Nanofertilizer Enhances Growth and Productivity of Capsicum annuum L
Source: Plants (Basel). 2021 Jun 4;10(6):1144. doi: 10.3390/plants10061144 (PMC8227464; doi:10.3390/plants10061144)
Supplement: Supplementary file 1 [file plants-10-01144-s001.zip › plants-1232957-supplementary.pdf]

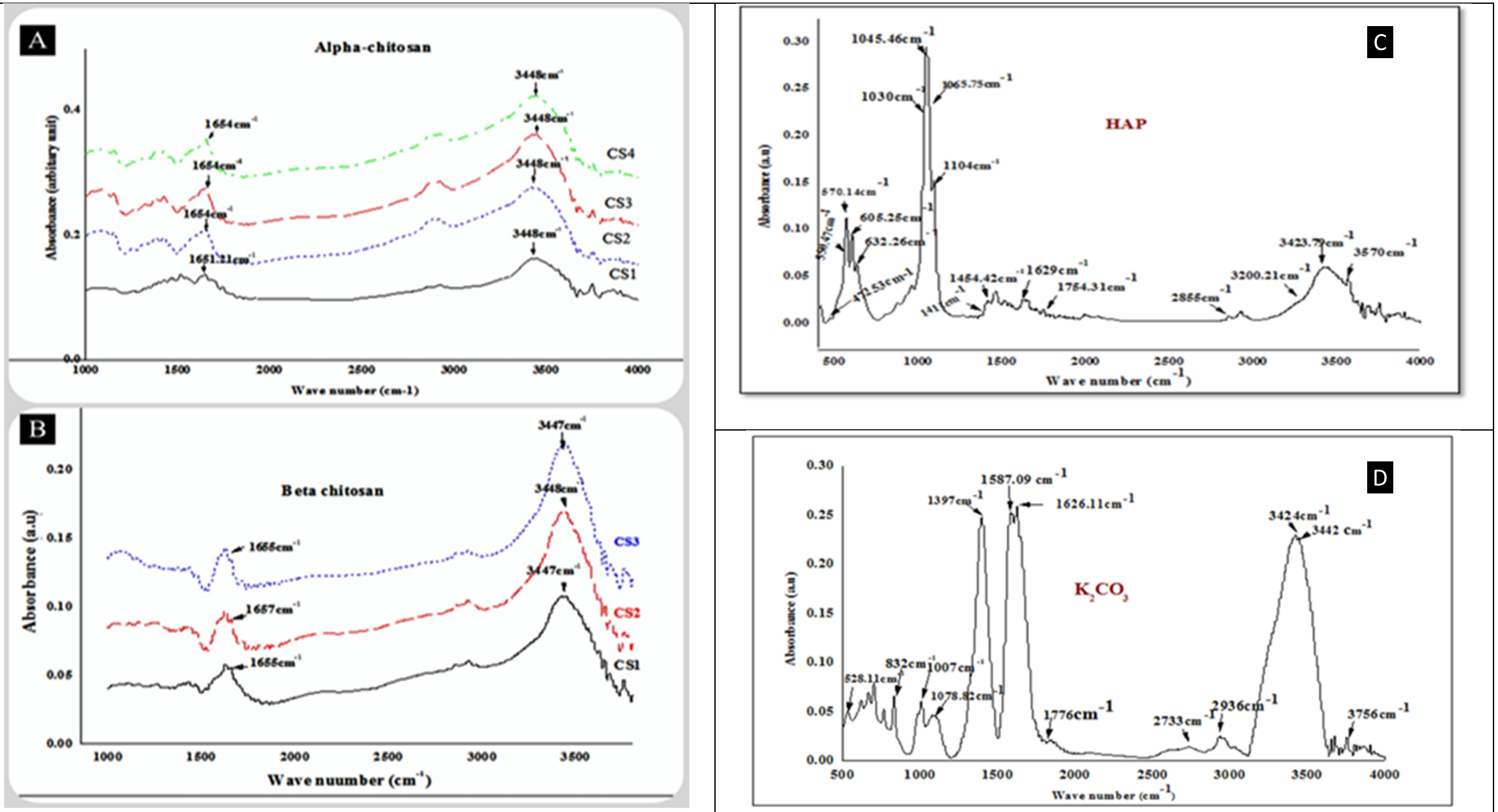

Figure S1. FT-IR spectra of α-CS (A), β-CS (B), (C) HAP and (D) PC obtained from recycled solid waste.

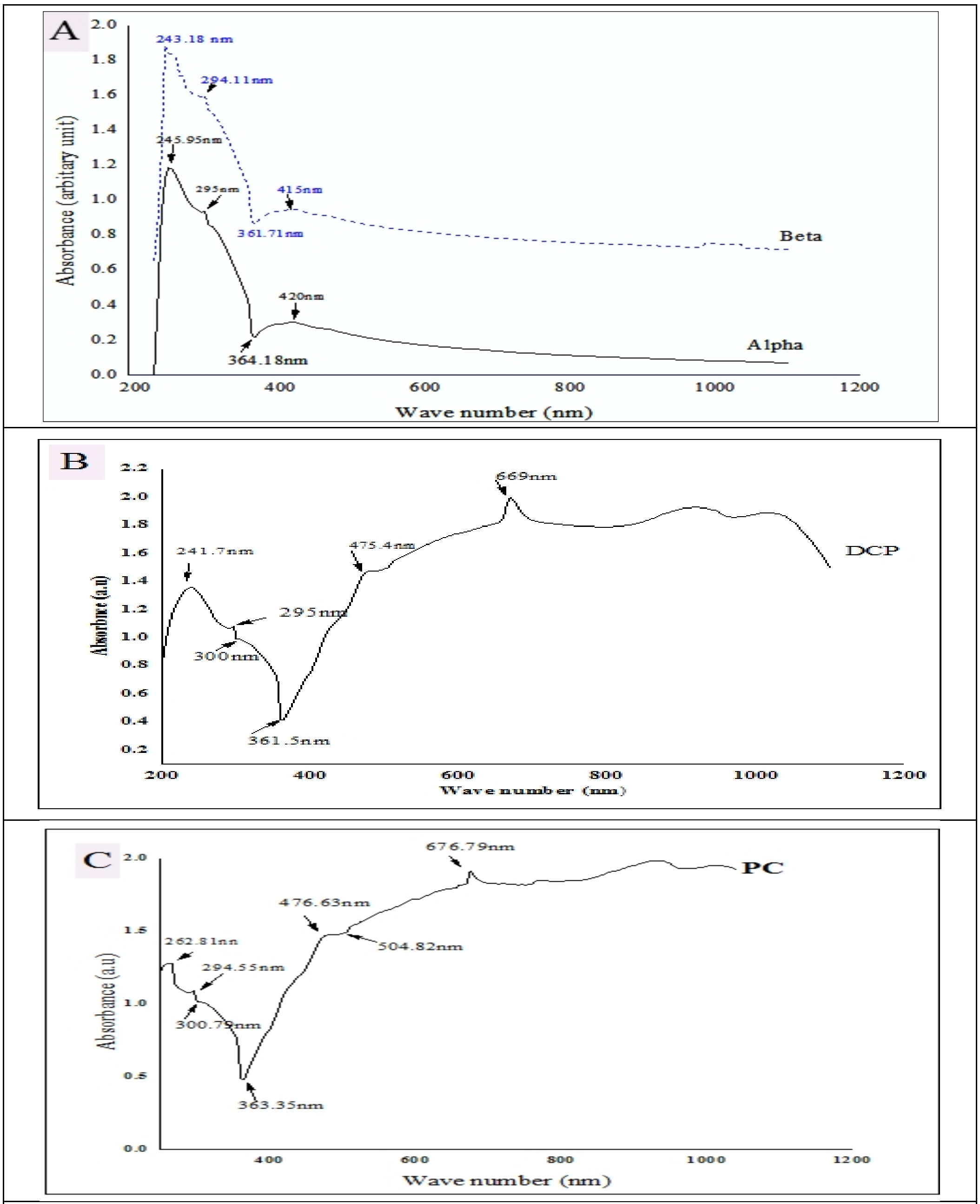

Figure S2. UV-spectra of the prepared nano-waste fertilizers, (A) α- and β-Chitosan, (B) dicalcium phosphate, and (C) Potassium

**Table S1:Wave length (cm^{-1}) of the main bands obtained for the prepared α- and β- Chitosans.**							
**Vibration modes**	**α-CS1**	**α-CS2**	**α-CS3**	**α-CS4**	**β-CS1**	**β-CS2**	**β-CS3**
NH out – of – plane bending	752	752	752	742	751	752	752
Ring stretching (CH bending out)	895	897	897	897	897	894	897
CO stretching	1067	1035	1035	1034	1033	1032	1033
C-O-C bridge	1156	1154	1153	1154	1157	1164	1164
C-N stretching of amide III	1321	1322	1321	1321	1321	1318	1318
	1343	1344	1344	1341	1347	1344	1344
CH_2 bending and CH_3 deformation	1427	1425	1424	1424	1435	1432	1432
Amide II band (N-H bending)	1545	1524	1523	1459	1469	1522	1522
Amide I band	1651.21	1654	1654	1654	1655	1657	1655
CH stretching	2860	2886	2886	2886	2887	2857	2857
Symmetric CH_3 stretching and asymmetric CH_2 stretching	2925	2923	2922	2923	2927	2926	2927
OH stretching	3448	3448	3447	3448	3447	3448	3447
Wave length (cm^{-1}) of the main bands obtained for the prepared K_2CO_3.							
Symmetric stretch of free carbonates(polydentate)	1078.82						
Free carbonates bending mode	702 and 823						
Free bicarbonates	1587.09						
Symmetric stretching mode of bidentate carbonates(C=O)	1626.11						
Symmetric bending mode of bidentate carbonates(C=O)	1776						
Symmetric bending mode of monodentate carbonate (C-O)	1397						
OH- group stretching	3424-3442						
Wave length (cm-1) of the main bands obtained for the prepared HAP..							
O—P—O asymmetric bending	570.14 and 605.25						
Asymmetric PO stretching	1030						
H2O absorbed by the lattice	3200-3570						
Free CO_3^{2-}	1629						
OH- group bending	632.26						
OH- group stretching	3570						
PO43- (free ion) stretching mode	1045						
P-O-- stretching	1104						
Fatty acids	1754.31						
Carbonated HAP	1411-1454.42						

Table S2: Particle size (nm) of prepared waste nano particles.	
Waste Nano-Particles name	Particle size (nm)±S.E
α-CS-NPs	17.41±1.86
β-CS-NPs	14.20±2.03
DCP-NPs	12.71±0.28
$K2CO_3$-NPs	28.30±2.21
